# Dose responses of vitamin D_3_ supplementation on arterial stiffness in overweight African Americans with vitamin D deficiency: A placebo controlled randomized trial

**DOI:** 10.1371/journal.pone.0188424

**Published:** 2017-12-07

**Authors:** Anas Raed, Jigar Bhagatwala, Haidong Zhu, Norman K. Pollock, Samip J. Parikh, Ying Huang, Robyn Havens, Ishita Kotak, De-Huang Guo, Yanbin Dong

**Affiliations:** 1 Georgia Prevention Institute, Department of Population Health Sciences, Medical College of Georgia, Augusta University, Augusta, Georgia, United States of America; 2 Department of Medicine, Medical College of Georgia, Augusta University, Augusta, Georgia, United States of American; 3 Department of Pediatrics, Medical College of Georgia, Augusta University, Augusta, Georgia, United States of America; Indiana University Richard M Fairbanks School of Public Health, UNITED STATES

## Abstract

**Background:**

Clinical trials are scant and equivocal on whether vitamin D can ameliorate arterial stiffness, particularly in populations at high risk for vitamin D deficiency and cardiovascular disease (CVD). This study determined the dose-response effects of vitamin D_3_ supplementation on arterial stiffness in overweight African Americans with vitamin D deficiency.

**Methods:**

Seventy overweight African Americans (aged 13–45 years) with serum 25-hydroxyvitamin D [25(OH)D] levels ≤ 20 ng/mL were randomized to monthly oral supplementation of 18,000 IU (~600 IU/day, *n* = 17), 60,000 IU (~2000 IU/day, *n* = 18), or 120,000 IU (~4000 IU/day, *n* = 18) of vitamin D_3_ or placebo (*n* = 17) for 16-weeks. The arterial stiffness measurements, carotid-femoral pulse wave velocity (PWV) and carotid-radial PWV, were assessed by applanation tonometry at baseline and 16 weeks.

**Results:**

Vitamin D_3_ supplementation demonstrated a dose-response increase in serum 25(OH)D concentrations between groups (*P*<0.01). A significant downward linear trend was observed for carotid-femoral PWV (*P*<0.01), as the mean changes in carotid-femoral PWV across the four treatment groups were 0.13 m/s (95% CI: -0.24, 0.51 m/s) for placebo, 0.02 m/s (95% CI: -0.34, 0.38 m/s) for 600 IU/day group, -0.11 m/s (95% CI: -0.50, 0.27 m/s) for the 2,000 IU/day group, and -0.70 m/s (95% CI: -1.07, -0.32 m/s) for the 4,000 IU/day group. Findings were similar for carotid-radial PWV (*P* = 0.03), as the mean changes in carotid-radial PWV across the four treatment groups were 0.24 m/s (95% CI: -0.45, 0.92 m/s) for placebo, 0.09 m/s (95% CI: -0.54, 0.73 m/s) for 600 IU/day group, -0.57 m/s (95% CI: -1.20, 0.07 m/s) for the 2,000 IU/day group, and -0.61 m/s (95% CI: -1.25, 0.02 m/s) for the 4,000 IU/day group.

**Conclusion:**

Arterial stiffness was improved by vitamin D_3_ supplementation in a dose-response manner in overweight African Americans with vitamin D deficiency.

## Introduction

African-Americans are prone to suboptimal vitamin D status [1]. In the last decade, vitamin D deficiency has been related to cardiovascular risk factors [2], cardiovascular mortality [3], stroke [4], and peripheral arterial disease [5]. Cardiovascular disease (CVD) is the leading cause of death among African Americans [6], which requires finding a tool for early recognition of pathological processes, diagnosis, prevention, and treatment [7]. Arterial stiffness is an independent predictor of CVD events and mortality [8, 9]. Arterial stiffness is not only a risk factor for CVD, but also a pathological mechanism leading to CVD through its influence on blood vessel and heart. In vessels, high shear stress in a stiff arterial system leads to amplification of endothelial dysfunction [10, 11]. Also, productivity of heart ejection and cardiac perfusion are impacted in individual with stiff arteries [12]. Pulse wave velocity (PWV) is a noninvasive, gold standard for arterial stiffness measurement, and a tool to determine the therapeutic effects and efficacy in interventional studies [13] [14]. Emerging evidence shows that vitamin D deficiency has been correlated with arterial stiffness [15, 16]. This suggests that investigating the impact of vitamin D supplementation on arterial stiffness is critical to reduce the cardiovascular morbidity and mortality.

Although vitamin D interventions are scant and equivocal on whether supplemental vitamin D can ameliorate arterial stiffness [17, 18], we previously observed that 2000 IU/day of vitamin D_3_ supplementation counteracted the progression of arterial stiffness in African-American adolescents [19]. More recently, we conducted another 16-week randomized placebo-controlled vitamin D_3_ supplementation trial (placebo, 600 IU/day, 2,000 IU/day, vs. 4,000 IU/day) in apparently healthy overweight African Americans with vitamin D deficiency, which revealed a dose-response increase in serum 25-hydroxyvitamin D [25(OH)D] concentrations [20]. The objective of this study was to perform a secondary analysis of this previously conducted randomized controlled trial to test the hypothesis that vitamin D_3_ supplementation reduces arterial stiffness in a dose-response manner.

## Materials and methods

### Study population and design

This study is an ancillary analysis to a previously completed, randomized, double-blinded, vitamin D_3_ supplementation trial (clinicaltrials.gov registration#: NCT01583621) [20]. The 70 participants in this trial were recruited from the Augusta, Georgia area by advertisements and by word of mouth. Inclusion criteria were the following: 1) African American race; 2) aged between 13 and 45 years; 3) overweight/obese [defined by body mass index (BMI) ≥25 kg/m^2^ for adults and ≥85^th^ percentile for age and sex for adolescents according to the Center of Disease Control and Prevention]; and 4) suboptimal vitamin D status (defined as serum 25(OH)D concentrations of ≤20 ng/mL at screening visit) [21]. Participants were excluded if they were pregnant or if they were taking medications/dietary supplements or had any medical condition that could affect nutritional status or metabolism. Adolescent participants and their guardian provided written informed assent and consent, respectively; and the adult participants provided informed consent. The study was approved by the Augusta University (AU) Institutional Review. All measurements were performed at the Medical College of Georgia’s Georgia Prevention Institute at Augusta University between December 2011 and November 2012. The trial registration on clinicaltrials.gov was delayed due to an oversight, and the authors confirm that all ongoing and related trials for this intervention are registered.

### Randomization and treatment allocation

**Fig 1:**
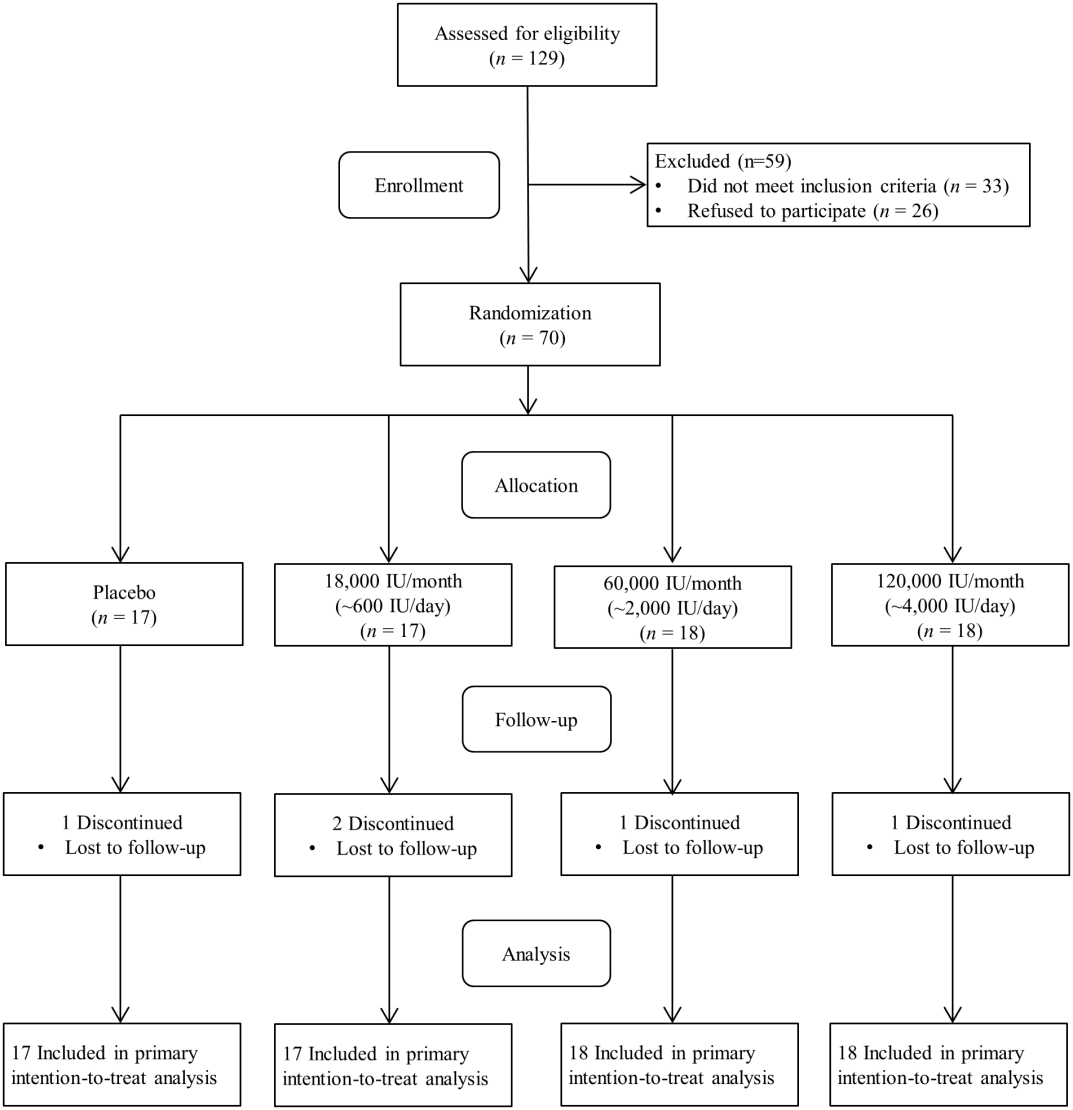
Flow diagram of study participants.

The CONSORT diagram (Fig 1) which was reported in previous publication gives the details of participant enrollment [20]. The recruited subjects were randomly assigned to four groups: 18,000 IU/month (~600 IU/day), 60,000 IU/month (~2,000 IU/day), or 120,000 IU/month (~4,000 IU/day) of vitamin D_3_ or placebo. The placebo and vitamin D_3_ containing capsules, provided by the Bio-Tech Pharmacal (Fayetteville, AR), were similar in taste and appearance and were indistinguishable for participants and investigators. The capsules were provided to the participants by supervised monthly dosing for 16-weeks to ensure compliance. The AU clinical research pharmacy generated the randomization codes, dispensed the study capsules, and maintained the randomization codes until the end of the study and did not have any direct role in the data collection. Study investigators, research coordinators, and the study participants were blinded to treatment allocation.

### Serum 25-hydroxyvitamin D

Fasting blood samples were collected by a trained phlebotomist at baseline, 8 weeks, and 16 weeks, and the samples were stored at −80°C until assayed. Using samples collected from each time point, serum 25(OH)D was measured using an enzyme immunoassay (Immunodiagnostic Systems, Fountain Hills, AZ). The mean intra- and interassay coefficients of variation for serum 25(OH)D were 5.6 and 6.6%, respectively. Analytical reliability of serum 25(OH)D assays was further monitored through DEQAS (the Vitamin D External Quality Assessment Scheme).

### Blood pressure

At baseline and 16 weeks, systolic blood pressure and diastolic blood pressure measurements (Dinamap 1864SX; Criticon, Inc., Tampa, FL) were taken three times at 5, 7, and 9 minutes after a 10-minute relaxation period in a quiet temperature controlled room. The average was used to represent systolic blood pressure and diastolic blood pressure.

### Arterial stiffness

The arterial stiffness measurements, carotid-femoral PWV and carotid-radial PWV, were measured at baseline and 16 weeks. As recommended by the American Heart Association [14], carotid-femoral PWV was our primary outcome measurement of arterial stiffness, and it was measured in duplicate using the SphygmoCor system (AtCor Medical, Sydney, Australia) by sequentially recording electrocardiographic-gated carotid and femoral artery waveforms by applanation tonometry (Millar Instruments, Houston, TX). Using a segmometer, straight-line distance measurements were taken from the suprasternal notch to the carotid sampling site and from the suprasternal notch to the site where the femoral artery was measured. The time interval between the onset of femoral and carotid waveforms was determined as the mean from 10 consecutive cardiac cycles. Quality measurements were confirmed by the standard deviation of time intervals corresponding to the patient’s ECG and femoral and carotid artery waveforms. Standard deviations greater than 10% of the carotid-femoral PWV value were not accepted. The carotid-femoral PWV was calculated from the distance between measurement points (D, in meters) and the measured time delay (t, in seconds) between the peak of the ECG P-wave and the trough of a waveform as follows: carotid-femoral PWV = D/t (m/s). The same investigator who performed the PWV measurements at baseline and posttest was blinded to the treatments. The within-observer variability (mean difference ± SD of two measurements) was 0.08 ± 1.10 m/s for carotid-femoral PWV and 0.15 ± 1.01 m/s for carotid-radial PWV.

### Statistical considerations and analyses

Power calculations were computed based on primary outcomes 25(OH)D and carotid-femoral PWV. The computations are based upon the linear contrast approach for the main effect of treatment, rather than the traditional omnibus *F*-test followed by post hoc pairwise comparisons, because a pre-specified contrast conditioned on the ordering of responses is more powerful to detect the expected pattern, without sacrificing control of type I error. Required are assumptions concerning the difference in mean 16-week changes in the response (i.e., differences in the mean gain scores) across treatment arms, and the variance of the gain score, from which effect size is computed. Using data previously collected in our laboratory [19, 22], we pooled estimates in standard deviation (SD) of change for 25(OH)D (SD = 5.1 ng/mL), and carotid-femoral PWV (SD = 0.8 m/s), and we estimated effect sizes (i.e., Cohen’s *d*) for these outcomes [25(OH)D, *d* = 3.0; and carotid-femoral PWV, *d* = 1.1] based on the assumption that the 600 IU and 2000 IU groups would have one-quarter and one-half the mean change, respectively, of that occurring in the 4000 IU group, relative the mean change in the control group. We determined that 10–16 subjects/group would provide 81–96% power (α = 0.05) to detect a difference in mean change of the outcome variable between groups. With a given sample size of 16 subject/group (a-level set at 0.05), the proposed study will have power of 0.81. Assuming a 10% sample size loss due to either attrition or insufficient quality of measurements, a starting sample size of 18 subjects/group (*N* = 72) will preserve power of the study design.

Characteristics of the participants at baseline were descriptively compared among the dose groups, with data presented as means ± SD if not stated otherwise. Group differences at baseline were determined by analysis of variance for normally distributed variables or by Kruskal-Wallis test, otherwise. Group differences in proportions at baseline were tested by χ^2^ test of goodness of fit.

Repeated-measures mixed models were used with restricted maximum likelihood estimation in an intention-to-treat analysis of each outcome measure using all available data. In the base models for carotid-femoral PWV, carotid-femoral PWV, and blood pressure, participant was included as a random effect, and intervention group (placebo, 600 IU/day, 2,000 IU/day, and 4,000 IU/day) and measurement time (baseline and 16 weeks) and their interaction were included as fixed effects. In serum 25(OH)D base models, fixed effects for measurement time had three levels (baseline, 8 weeks, and 16 weeks) since it was also measured at 8 weeks. Covariance structures were compared by using the Akaike information criterion; the autoregressive structure and the compound symmetry structure had similar Akaike information criterion values, so the compound symmetry structure was chosen. In addition, we adjusted for age, gender, BMI, and season in a secondary analysis to assess their contribution to the changes in the arterial stiffness measurements associated with the intervention. *A priori* linear contrasts tested dose-response effects of the intervention. If the trend for difference in the outcome variable of interest across groups was significant (*P* < 0.05), differences among individual groups, adjusted for multiple comparisons, were tested by using Tukey’s honestly significant difference adjustment.

Model fit was examined by looking at various residual plots. A sensitivity analysis was performed by using multiple imputation to determine whether the missing data affected the carotid-femoral PWV and carotid-femoral PWV. Group membership and measurements over time were used in the imputation process, and the imputations were conducted using two methods. On the basis of available outcome data from all randomized participants, an initial imputation based on Markov Chain Monte Carlo algorithm was used to establish a monotone missing data pattern. Missing values in the monotone data set were subsequently imputed multiple times (5 imputations) in a second step using regression procedures [23]. The week 16 outcome measurements were calculated using the imputed data sets and analyzed using analysis of variance. The data from the five analyses were subsequently combined into single estimates and tested as described by Schafer [24]. All statistical analyses were performed with the use of SPSS software (version 24, IBM SPSS Statistics, Chicago, IL) and SAS software (version 9.4, SAS Institute, Cary, NC), and statistical significance was set at *P* < 0.05.

## Results

The flow diagram is presented in Fig 1. Of 129 screened, ~75% (96 individuals) met the inclusion criteria. A total of 70 subjects were available at baseline. Sixteen percent of the total sample were males, and 79% were obese. The baseline mean serum 25(OH)D concentration in the total sample was 14.0 ± 4.0 ng/mL. None of the participants reported side effects. The baseline characteristics for the vitamin D and placebo groups are shown in Table 1. The four groups did not differ in sex distribution, age, body mass index, season of study visit, serum 25(OH)D, blood pressure, carotid-femoral PWV, or carotid-radial PWV at baseline.

**Table 1:** Baseline participant characteristics.

	Placebo	600 IU/day	2,000 IU/day	4,000 IU/day	*P*-value*
*n*	17	17	18	18	
Male sex [*n* (%)]^†^	4 (24)	2 (12)	3 (17)	2 (11)	0.46
Age (y)	27.8 ± 9.9	26.2 ± 9.8	24.4 ± 8.7	25.5 ± 9.0	0.77
Body mass index (kg/m^2^)	36.2 ± 8.3	34.6 ± 5.4	37.1 ± 8.0	34.4 ± 7.2	0.66
Season [*n* (%)]^†^					0.51
Winter	9(53)	9(53)	9(50)	11(61)	
Spring	8(47)	6(35)	8(44)	4(22)	
Summer	0(0)	2(12)	1(5)	3(17)	
Fall	0 (0)	0 (0)	0 (0)	0 (0)	
Serum 25-hydroxyvitamin D (ng/mL)	15.9 ± 3.9	14.0 ± 3.9	15.9 ± 3.7	13.3 ± 4.4	0.21
Systolic blood pressure (mm Hg)	126 ± 15	126 ± 16	126 ± 10	126 ± 13	0.98
Diastolic blood pressure (mm Hg)	65 ± 10	66 ± 12	66 ± 8	64 ± 10	0.95
Pulse wave velocity (m/s)					
Carotid-femoral	6.11 ± 1.12	7.09 ± 1.89	6.13 ± 1.00	6.71 ± 1.41	0.71
Carotid-radial	7.47 ± 1.20	7.53 ± 1.36	7.72 ± 1.62	7.60 ± 1.44	0.89

Values for categorical variable is given as number (percentage); values for continuous variables, as means ± SD.

*Test of significance between groups were based on analysis of variance.

^†^Test of significance between groups were based on chi-square test.

### Effect of vitamin D supplementation on 25(OH)D at 8- and 16-weeks

**Fig 2:**
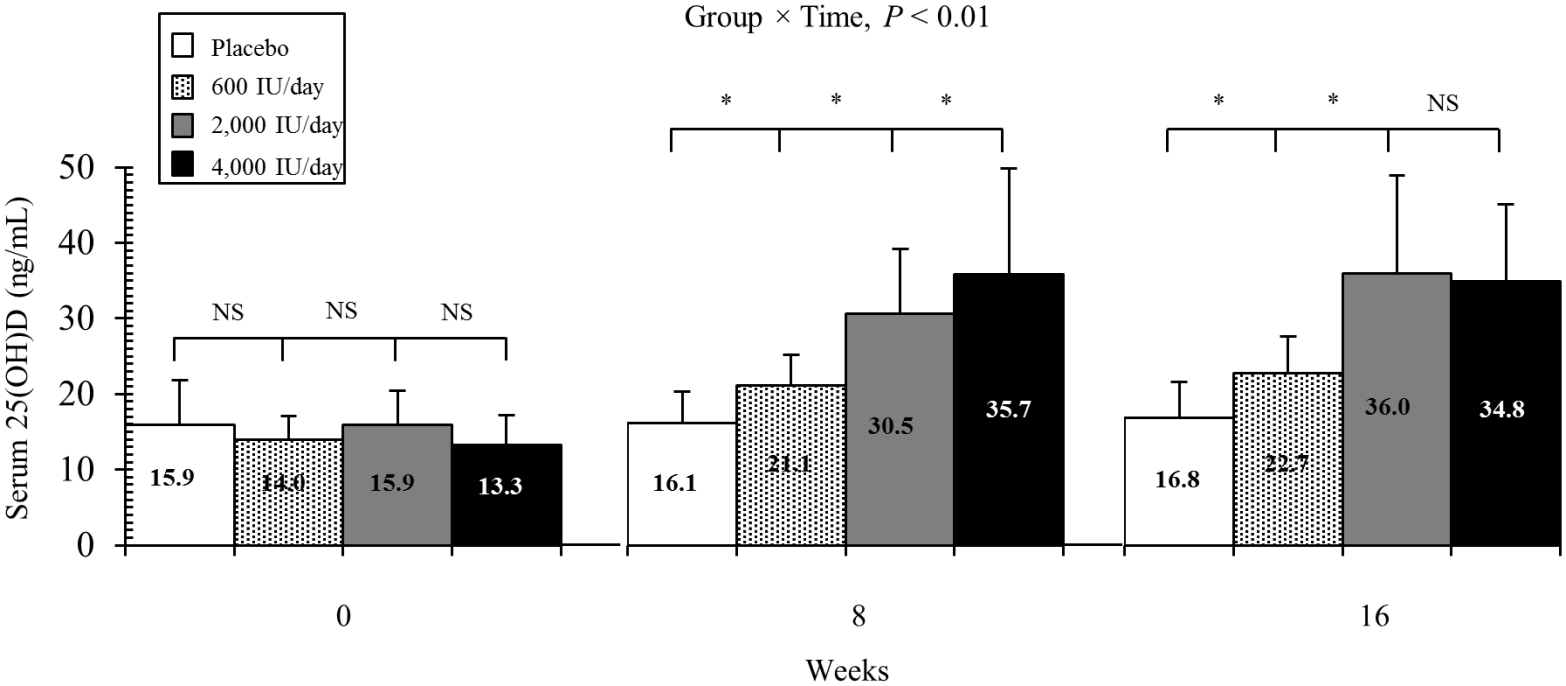
Serum 25-hydroxyvitamin D [25(OH)D] concentrations at baseline, 8 weeks, and 16 weeks. Response to 16 weeks of monthly supplementation in the form of either placebo (*n* = 17), 18,000 IU of vitamin D_3_ (600 IU/day, *n* = 17), 60,000 IU of vitamin D_3_ (2,000 IU/day, *n* = 18), or 120,000 IU of vitamin D_3_ (4,000 IU/day, *n* = 18). Values are means ± SD. The *P* value indicates the test of the dose-response trend. Data represent mean ± SD. *P < 0.05, NS = not significant.

Fig 2 shows the mean serum 25(OH)D concentrations at 8 weeks and 16 weeks in response to 16 weeks of monthly supplementation in the form of either placebo or 18,000 IU (600 IU/day), 60,000 IU (2,000 IU/day), or 120,000 IU of vitamin D_3_ (4,000 IU/day). As previously reported [20], changes over time of serum 25(OH)D concentrations were significantly different between treatment groups (group x time, *P* < 0.01). After 8 weeks of supplementation with 600 IU/day vitamin D_3_, serum 25(OH)D concentrations increased 50%, while after 16 weeks the mean 25(OH)D concentrations increased slightly further to 61.4%. In the 2,000 IU/day group, the mean 25(OH)D concentrations increased 91.8% and 126.4%, respectively, at 8 weeks and 16 weeks. The highest level of percentage change in 25(OH)D concentrations after 8 weeks (168.4%) was observed in the 4,000 IU/day group. However, after 16 weeks in the 4,000 IU/day group, the mean 25(OH)D concentrations reached a plateau and did not increase any further (161.7%). Post hoc comparisons showed that changes in 25(OH)D concentrations were significantly greater in the 4,000 IU group vs. 2,000 IU group after 8 weeks (*P* < 0.01), but not after 16 weeks (*P* = 0.61).

### Effect of vitamin D supplementation on arterial stiffness at 16 weeks

**Fig 3:**
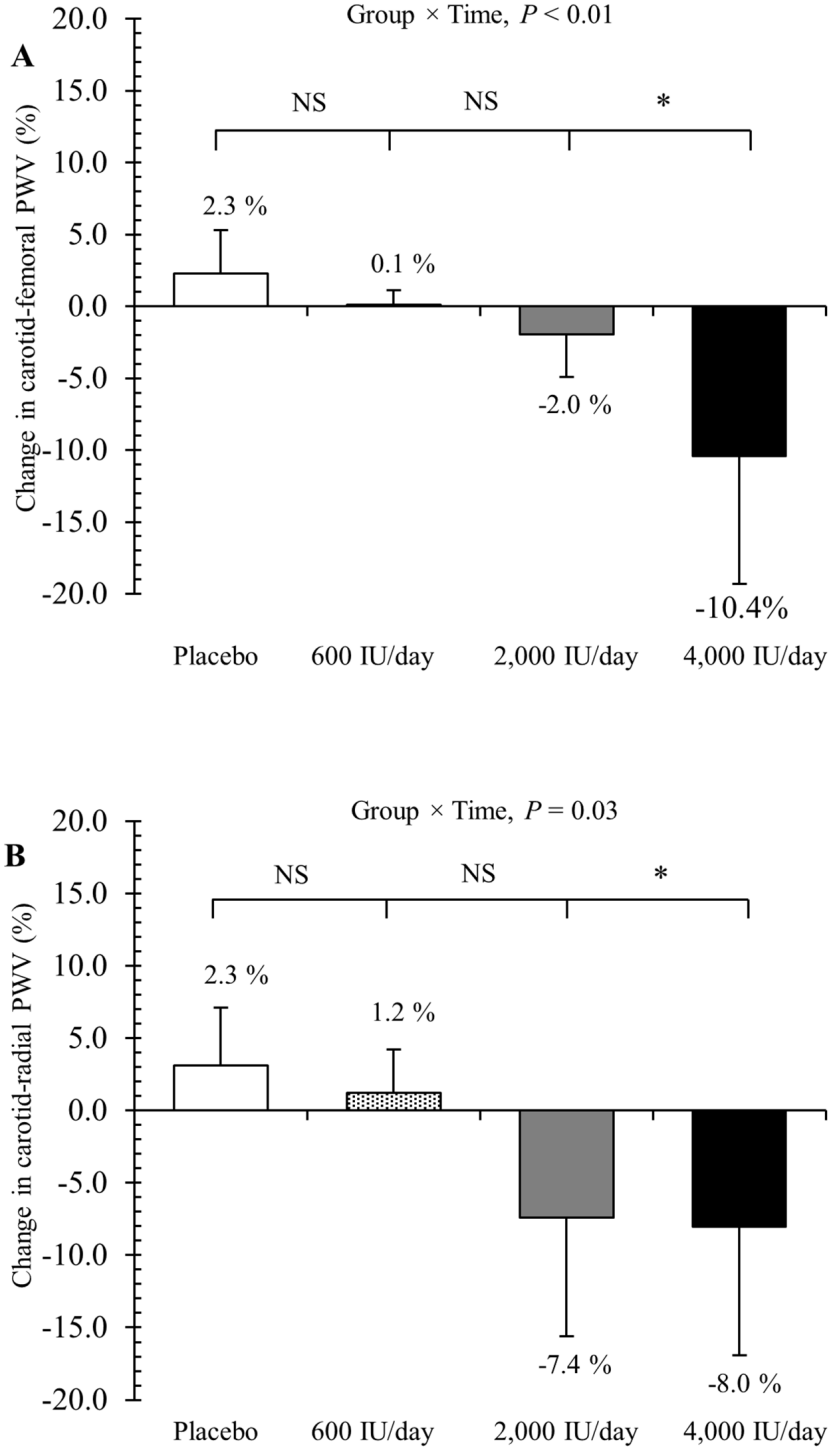
Effect of vitamin D_3_ supplementation on arterial stiffness. Relative changes from baseline in carotid-femoral pulse wave velocity (A) and carotid-radial pulse wave velocity (B) in response to 16 weeks of monthly supplementation in the form of either placebo (*n* = 17), 18,000 IU of vitamin D_3_ (600 IU/day, *n* = 17), 60,000 IU of vitamin D_3_ (2,000 IU/day, *n* = 18), or 120,000 IU of vitamin D_3_ (4,000 IU/day, *n* = 18). The *P*-value indicates the test of the dose-response trend. Data represent mean ± SD. *P < 0.05, NS = not significant.

The effect of the various doses of vitamin D_3_ supplementation on arterial stiffness measurements after 16 weeks are shown in Table 2 and Fig 3. In the primary mixed model analysis, a significant downward linear trend was observed for carotid-femoral PWV (group x time, *P* < 0.01) (Table 2 and Fig 3A), as the mean changes in carotid-femoral PWV across the four treatment groups were 0.13 m/s (95% CI, -0.24 to 0.51 m/s) for placebo, 0.02 m/s (95% CI, -0.34 to 0.38 m/s) for 600 IU/day group, -0.11 m/s (95% CI, -0.50 to 0.27 m/s) for the 2,000 IU/day group, and -0.70 m/s (95% CI, -1.07 to -0.32 m/s) for the 4,000 IU/day group. Likewise, a significant downward linear trend was observed for carotid-radial PWV (group x time, *P* = 0.03) (Table 2 and Fig 3B), as the mean changes in carotid-radial PWV across the four treatment groups were 0.24 m/s (95% CI, -0.45 to 0.92 m/s) for placebo, 0.09 m/s (95% CI, -0.54 to 0.73 m/s) for 600 IU/day group, -0.57 m/s (95% CI, -1.20 to 0.07 m/s) for the 2,000 IU/day group, and -0.61 m/s (95% CI, -1.25 to 0.02 m/s) for the 4,000 IU/day group. Post hoc comparisons showed that the decreases in carotid-femoral PWV (-10.4% vs. -2.0%, respectively) and carotid-radial PWV (-8.0 vs. -7.4%, respectively) from baseline to 16-weeks were significantly greater in the 4000 IU/day group vs. 2000 IU/day group (both *P* ≤ 0.05). The results of the sensitivity analysis using multiple imputation models were similar to the missing-completely-at-random mixed model analysis (S1 Data).

**Table 2:** Arterial stiffness measurements at baseline and 16 weeks in overweight African Americans with vitamin D deficiency randomly assigned to 16 weeks of monthly supplementation in the form of either placebo (*n* = 17), 18,000 IU of vitamin D_3_ (600 IU/day, *n* = 17), 60,000 IU of vitamin D_3_ (2,000 IU/day, *n* = 18), or 120,000 IU of vitamin D_3_ (4,000 IU/day, *n* = 18).

	Baseline	16 weeks	Change	Group x time, *P*-value*
Carotid-femoral PWV (m/s)				<0.01
Placebo	6.11 (5.35 to 6.88)	6.25 (5.49 to 7.00)	0.13 (-0.24 to 0.51)	
600 IU/day	7.09 (6.35 to 7.82)	7.10 (6.38 to 7.83)	0.02 (-0.34 to 0.38)	
2,000 IU/day	6.13 (5.33 to 6.92)	6.01 (5.22 to 6.80)	-0.11 (-0.50 to 0.27)	
4,000 IU/day	6.71 (5.94 to 7.48)	6.01 (5.26 to 6.77)	-0.70 (-1.07 to -0.32)	
Carotid-radial PWV (m/s)				0.03
Placebo	7.47 (6.64 to 8.29)	7.70 (6.95 to 8.46)	0.24 (-0.45 to 0.92)	
600 IU/day	7.53 (6.76 to 8.29)	7.62 (6.93 to 8.32)	0.09 (-0.54 to 0.73)	
2,000 IU/day	7.72 (6.96 to 8.49)	7.15 (6.46 to 7.85)	-0.57 (-1.20 to 0.07)	
4,000 IU/day	7.60 (6.84 to 8.37)	6.99 (6.29 to 7.68)	-0.61 (-1.25 to 0.02)	

Values are means (95% CI). PWV, pulse wave velocity.

*P-value indicates the test of the dose-response trend.

In a secondary analysis adjusting for age, gender, BMI and season of study visit, the findings for carotid-femoral PWV and carotid-radial PWV were similar to the models without covariates (S2 Data). When we assessed the effect of vitamin D_3_ supplementation on blood pressure after 16 weeks, there were no significant changes in systolic BP (*P* = 0.86) or diastolic BP (*P* = 0.83) between treatment groups.

## Discussion

To our knowledge, this is the first randomized controlled trial regarding dose-ranging vitamin D supplementation on measures of arterial stiffness in overweight African-Americans with vitamin D deficiency. The key finding from this study was that arterial stiffness was improved by vitamin D_3_ supplementation in a dose-response manner.

It is hypothesized that vitamin D deficiency is linked to greater arterial stiffness, suggesting that increasing vitamin D intake may improve arterial stiffness [15]. Few randomized clinical trials, to date, have assessed the effect of vitamin D supplementation on arterial stiffness measured by PWV. Among the vitamin D and arterial stiffness clinical trials, the results have been inconsistent [17]. Discrepancies may be attributed in part to differences in study design and instruments used. Studies were unsuccessful in increasing vitamin D to the optimal range due to giving one single dose of vitamin D at baseline [25], or utilizing low dose (<2000 IU/day) [25–27].

Other possible reasons for study inconsistencies may be due, in part, to differences in study duration, vitamin D administration, the vitamin D status of the study participants before and after intervention, and adherence to the supplements. Marckmann et al. [28] showed no effect of vitamin D on biomarkers of CVD including PWV, which could be attributed to the short follow-up time (8 weeks). In the study by McGreevy and colleagues [25], their vitamin D intervention was a relative short duration of 8-weeks; the vitamin D supplement was administered intramuscularly rather than orally, which has better bioavailability [29]; and participants were still vitamin D deficient (<20 ng/mL) after the intervention. It is postulated that, in vitamin D deficient individuals, 12-weeks of vitamin D_3_ supplementation is needed to achieve optimal vitamin D status [30]. Additionally, Larsen et al. [18] and Gepner et al. [31] reported that PWV was not reduced probably due to recruiting participants with optimal vitamin D levels at baseline.

African-Americans have lower vitamin D levels and greater arterial stiffness [1, 32, 33]. It is noteworthy that African-Americans have higher CVD morbidity and mortality [6], which may be attributed to both arterial stiffness and vitamin D deficiency. The mechanism for beneficial effects of vitamin D on arterial stiffness is unknown. However, one potential pathway is via the renin-angiotensin-aldosterone system. For instance, animal studies using the vitamin D receptor knock-out mouse have revealed that both high renin expression and angiotensin II synthesis can increase vascular tone and arterial stiffness [34, 35]. Other potential hypotheses include vitamin D’s ability to suppress endothelin-induced vascular smooth muscle cell proliferation, macrophages activation, and vascular calcifications [15]. Also, vitamin D has immunomodulatory properties which modulate the acquired immune system by reducing inflammation, suppressing T-cell proliferation, and reducing inflammatory cytokines [36]. In this study and our prior investigation in African Americans [19], vitamin D supplementation of 2,000 or 4,000 IU/day reduced arterial stiffness, as measured by carotid-femoral PWV. Another important finding in our study was that the decrease in carotid-femoral PWV was significantly greater in the 4,000 IU/day group (-10.4%) compared to the 2,000 IU/day group (-2.0%). This could be explained by the immunomodulatory capacity of different doses of vitamin D as studies showed dose dependent responses of vitamin D on reducing inflammation and cytokines synthesis [37–39]. It is important to note, however, that the mean serum 25(OH)D concentrations after 16 weeks of supplementation were similar between the 4,000 IU/day (34.8 ng/mL) and 2,000 IU/day (36.0 ng/mL) groups. It is possible that the greater decrease in carotid-femoral PWV observed in the 4,000 IU/day group vs. 2,000 IU/day group after 16 weeks may reflect the higher serum 25(OH)D concentrations detected in the 4,000 IU/day group (35.7 ng/mL) vs. 2,000 IU/day group (30.5 ng/mL) after 8 weeks. Therefore, it is plausible that circulating 25(OH) concentrations may need to be maintained at ~35 ng/mL or higher for longer than 8 weeks in order for advantageous effects on arterial stiffness can be observed.

Our study has several strengths and limitations that need to be discussed. Strengths include the following: double blind randomized controlled study design, supervised monthly dosing administration to achieve 100% compliance, and the doses selected in this study were based on the current Recommended Dietary Allowance (600 IU/day) and the tolerable upper intake level (4,000 IU/day) by the Institute of Medicine [21]. Limitations should be acknowledged. First, our sample size was relatively small, large RCTs are warranted to validate the findings. Second, the participant recruitment was done in different seasons. However, we recruited participants with suboptimal vitamin D status across all seasons and the results were not altered after adjusting for seasons. Finally, the female distribution was higher in our sample compared to male. However, the gender representation was not different among groups, and the result did not differ after adjusting for gender as confounder.

In conclusion, vitamin D_3_ supplementation improves arterial stiffness in a dose-response manner in overweight African-Americans with vitamin D deficiency. Larger trials are needed to determine whether vitamin D_3_ supplementation reduces the risk of CVD events in at risk populations.

## Supporting information

S1 Data Unadjusted mean changes from baseline in arterial stiffness measurements in response to vitamin D supplementation in 4 groups. (DOCX)

S2 Data Adjusted mean changes from baseline in arterial stiffness measurements in response to vitamin D supplementation in 4 groups. (DOCX)
