# Supplementary material for: Dose responses of vitamin D3 supplementation on arterial stiffness in overweight African Americans with vitamin D deficiency: A placebo controlled randomized trial
Source: PLoS One. 2017 Dec 7;12(12):e0188424. doi: 10.1371/journal.pone.0188424 (PMC5720756; doi:10.1371/journal.pone.0188424)
Supplement: S1 Data — (DOCX) [file pone.0188424.s001.docx]

**S1 Table.** Unadjusted mean (95% CI) changes from baseline in arterial stiffness measurements in response to 16 weeks of monthly supplementation of either placebo, 18,000 IU vitamin D_3_ (600 IU/day), 60,000 IU vitamin D_3_ (2,000 IU/day), or 120,000 IU vitamin D_3_ (4,000/day)

|  | Placebo | 600 IU/day | 2,000 IU/day | 4,000 IU/day | Group x Time, *P*-value* | |
| --- | --- | --- | --- | --- | --- | --- |
| *n* | 17 | 17 | 18 | 18 |  | |
| Change in carotid-femoral PWV (m/s) |  |  |  |  | |  |
| - Mixed model analysis | 0.13 (-0.24 to 0.51) | 0.02 (-0.34 to 0.38) | -0.11 (-0.50 to 0.27) | -0.70 (-1.07 to -0.32) | | 0.005 |
| - Multiple imputation analysis | 0.13 (-0.22 to 0.48) | 0.02 (-0.39 to 0.42) | -0.11 (-0.54 to 0.32) | -0.70 (-1.10 to -0.29) | | 0.003 |
| Change in carotid-radial PWV (m/s) |  |  |  |  | |  |
| - Mixed model analysis | 0.24 (-0.45 to 0.92) | 0.09 (-0.54 to 0.73) | -0.57 (-1.20 to 0.07) | -0.61 (-1.25 to 0.02) | | 0.033 |
| - Multiple imputation analysis | 0.23 (-0.27 to 0.74) | 0.09 (-0.43 to 0.63) | -0.57 (-1.36 to 0.23) | -0.61 (-1.44 to 0.21) | | 0.032 |

Values are means (95% CI). PWV; pulse wave velocity.

^*^*P*-value indicates the test of the dose-response trend
