# Supplementary material for: Dose responses of vitamin D3 supplementation on arterial stiffness in overweight African Americans with vitamin D deficiency: A placebo controlled randomized trial
Source: PLoS One. 2017 Dec 7;12(12):e0188424. doi: 10.1371/journal.pone.0188424 (PMC5720756; doi:10.1371/journal.pone.0188424)
Supplement: S2 Data — (DOCX) [file pone.0188424.s002.docx]

**S2 Table.** Adjusted mean (95% CI) changes from baseline in arterial stiffness measurements in response to 16 weeks of monthly supplementation of either placebo, 18,000 IU vitamin D_3_ (600 IU/day), 60,000 IU vitamin D_3_ (2,000 IU/day), or 120,000 IU vitamin D_3_ (4,000/day)

|  | Placebo | 600 IU/day | 2,000 IU/day | 4,000 IU/day | Group x Time, *P*-value* |
| --- | --- | --- | --- | --- | --- |
| *n* | 17 | 17 | 18 | 18 |  |
| Change in carotid-femoral PWV (m/s) |  |  |  |  |  |
| - Mixed model analysis | 0.11 (-0.25 to 0.47) | 0.04 (-0.31 to 0.40) | -0.13 (-0.48 to 0.22) | -0.61 (-0.97 to -0.52) | 0.016 |
| - Multiple imputation analysis | 0.12 (-0.26 to 0.49) | 0.05 (-0.30 to 0.41) | -0.15 (-0.53 to 0.23) | -0.68 (-1.06 to -0.31) | <0.001 |
| Change in carotid-radial PWV (m/s) |  |  |  |  |  |
| - Mixed model analysis | 0.23 (-0.38 to 0.85) | 0.11 (-0.52 to 0.74) | -0.51 (-1.12 to 0.10) | -0.70 (-1.33 to 0.06) | 0.018 |
| - Multiple imputation analysis | 0.26 (-0.43 to 0.95) | 0.09 (-0.55 to 0.72) | -0.51 (-1.16 to 0.14) | -0.69 (-1.34 to 0.05) | 0.027 |

Values are adjusted means (95% CI). Means were adjusted for age, gender, body mass index, and season. PWV; pulse wave velocity.

**P*-value indicates the test of the dose-response trend.
